# Expression and prognostic value of transcription factor PROX1 in colorectal cancer

**DOI:** 10.1038/bjc.2011.297

**Published:** 2011-10-04

**Authors:** M Skog, P Bono, M Lundin, J Lundin, J Louhimo, N Linder, T V Petrova, L C Andersson, H Joensuu, K Alitalo, C H Haglund

**Affiliations:** 1 Department of Oncology, Helsinki University Central Hospital, Helsinki FIN-00029 HUS, Finland; 2 Molecular Cancer Biology Research Program, Biomedicum Helsinki, University of Helsinki, Helsinki FIN-00014 HY, Finland; 3 Department of Oncology, Institute of Clinical Medicine, University of Helsinki, Helsinki FIN-00014 HY, Finland; 4 Folkhälsan Research Centre, University of Helsinki, Helsinki FIN-00014 HY, Finland; 5 Institute for Molecular Medicine Finland, University of Helsinki, Helsinki FIN-00014 HY, Finland; 6 Department of Surgery, Helsinki University Central Hospital, Helsinki FIN-00029 HUS, Finland; 7 Department of Pathology, Haartman Institute, University of Helsinki, Helsinki FIN-00014 HY, Finland

**Keywords:** prospero-related homeobox gene, PROX1, colorectal cancer, expression, prognosis

## Abstract

**Background:**

PROX1 is a specific target of the *β*-catenin/TCF pathway in the intestinal epithelium. It acts as a regulator of progression from a benign to a highly dysplastic phenotype in colorectal tumours. However, the clinical significance of PROX1 expression is not known.

**Methods:**

We studied the prognostic value of immunohistochemical expression of PROX1 in a series of 517 patients with colorectal cancer (CRC).

**Results:**

The majority of the tumour samples expressed PROX1 (91%, 471 out of 517). High PROX1 expression was associated with a poor grade of tumour differentiation (*P*<0.0001). In the subgroup of patients with colon cancer, high PROX1 expression was associated with unfavourable colorectal cancer-specific survival (CCSS) as compared with low PROX1 expression (CCSS 47% *vs* 62% *P*=0.045; RR 1.47). The association between high PROX1 and poor outcome was further strengthened in female colon cancer patients (CCSS 38% *vs* 63% *P*=0.007; RR 2.02). Nonetheless, in multivariate survival analysis PROX1 expression was not retained as an independent prognostic factor.

**Conclusion:**

High PROX1 expression is associated with a poor grade of tumour differentiation, and, in colon cancer patients, also with less favourable patient outcome. Our results strengthen the previous preclinical observations that PROX1 has a role in tumour progression in CRC.

Colorectal cancer (CRC) is the third most commonly diagnosed cancer in the world (Parkin *et al*, 2005). Only a small fraction of CRCs occurs in dominantly inherited patterns. The two best-defined familial forms are familial adenomatous polyposis-related CRC and hereditary nonpolyposis colorectal cancer (reviewed in Kinzler and Vogelstein, 1996). Activation of the APC/*β*-catenin/TCF pathway is an initiating event of neoplasia in familial adenomatous polyposis patients. The *adenomatous polyposis coli* (*APC*) and *β-catenin* (*CTNNB1*) genes are two major components of the Wnt signalling pathway that are affected by mutations in CRC (Segditsas and Tomlinson, 2006). In normal cells, the APC protein binds to cytoplasmic *β*-catenin, targeting it for degradation. When the degradation is inhibited by Wnt signalling, *β*-catenin begins to accumulate in the nuclei of colorectal epithelial cells. Wnt signalling results in the formation of a complex containing *β*-catenin and T-cell factor (TCF). Familial adenomatous polyposis patients with *APC* mutation and blocked *β*-catenin degradation have an overactivated Wnt signalling pathway, which results in development of hundreds of intestinal polyps, and eventually CRC.

Loss of *APC* in the intestinal epithelium induces expansion of the progenitor cell population. The *β*-catenin/TCF pathway controls cancer cell proliferation and expression of progenitor cell-specific genes (Sansom *et al*, 2004). In humans, the progression from benign adenoma to malignant carcinoma takes several years and the cascade causing the malignant transformation is still unknown. Petrova *et al* (2008) have previously shown that transcription factor PROX1 is an intestinal specific target of the *β*-catenin/TCF pathway and has an essential role as a regulator of progression from a benign to a highly dysplastic phenotype in colorectal tumours.

PROX1 is an atypical homeodomain protein important for embryonic development of the lens, retina, liver, pancreas, and lymphatic vasculature, but little is known about PROX1 function in adult tissues (Oliver *et al*, 1993; Wigle and Oliver, 1999; Sosa-Pineda *et al*, 2002; Dyer *et al*, 2003). PROX1 is the mammalian homologue of the *Drosophila* homeobox protein Prospero, which acts as a brain tumour suppressor by inhibiting neuroblast self-renewal (Oliver *et al*, 1993; Betschinger *et al*, 2006). It has been suggested that PROX1 has a similar role in human cancer (Shimoda *et al*, 2006; Laerm *et al*, 2007). However, a recent publication shows that PROX1 expression is associated with a higher grade in astrocytic gliomas, the most common brain tumour type in adults (Elsir *et al*, 2010). Diverse results propose different roles for PROX1 in different cancer types. Moreover, the findings showing that PROX1 is overexpressed in the majority of CRCs and that it promotes neoplasia, tumour growth, and malignant progression suggest that PROX1 expression may be associated with the outcome of CRC patients (Petrova *et al*, 2008). In the present study, we investigated the clinical significance of PROX1 expression by immunohistochemistry in a large series of CRC patients.

## Patients and methods

### Patients

This study is based on a series of 643 consecutive patients who underwent surgery for histologically verified CRC at the Helsinki University Central Hospital in 1989–1998. The median follow-up time of the patients alive at the end of follow-up was 9 years (range 0.1–15.4). A tissue specimen suitable for evaluation of PROX1 expression by immunohistochemistry was available in 517 (80.4%) cases. Follow-up data, collected from the patient records and the files of the Finnish Cancer Registry and Statistics Finland, were available for all patients. The clinicopathological characteristics of the patients have been described previously in detail by Linder *et al* (2009), and are listed briefly in Table 1.

### Immunohistochemistry

PROX1 expression was assessed from tissue microarrays prepared as described in detail elsewhere (Linder *et al*, 2009). The tissue microarray blocks were cut into 4-*μ*m-thick sections, fixed on slides, and dried for 12–24 h at 37 °C. The sections were then deparaffinised in xylene and rehydrated through graded alcohol series. For antigen retrieval, the sections were heated in the Pretreatment Module of the Autostainer 480 (LabVision UK Ltd, Newmarket, UK) in Tris-EDTA buffer (pH 9.0) for 20 min at 98 °C. The staining of the sections was performed in Autostainer 480. The tissue sections were then treated with 0.3% Dako REAL Peroxidase-Blocking Solution (Dako Denmark A/S, Glostrup, Denmark) for 30 min to block the endogenous peroxidases, followed by incubation with rabbit normal serum (Vectastain ABC Kit, Vector, Burlingame, CA, USA) diluted 1:50 in TNB blocking solution (0.1 M Tris-HCl (pH 7.5), 0.15 M NaCl, 0.5% Blocking reagent (supplied in kit); Renaissance TSA Biotin System; Perkin-Elmer, Boston, MA, USA) for 30 min. Goat anti-PROX1 antibody (R&D Systems, Minneapolis, MN, USA) was used to detect PROX1 expression. The antibody was diluted 1:2000 in TNB blocking solution and incubated with the samples overnight at +4 °C. The tissue sections were then incubated for 1 h with a biotinylated anti-goat secondary antibody (Vectastain ABC Kit), diluted 1:300 in TNB blocking solution, and treated for 30 min with Strepavidin-HRP Conjugate (Perkin-Elmer) diluted 1:1250 in TNB blocking reagent. Immunostaining was visualised with Dako REAL Diaminobenzidine Chromogen (10 min treatment). After each step in the staining procedure, the slides were washed with wash buffer (137 mM NaCl, 10 mM phosphate, 2.7 mM KCl, 0.04% Tween 20; pH 7.4). Finally, the slides were counterstained with Meyer's haematoxylin, washed in tap water for 10 min, and mounted in Aquamount (BDH, Poole, UK). Specificity of the PROX1 immunopositivity was confirmed by staining the same tissue without the primary antibody.

### Scoring of PROX1 immunostaining

PROX1 expression was evaluated by two of the investigators (LCA and MS). Both the investigators were blinded to the clinicopathological data at the time of scoring. PROX1 staining in the cancer cell nuclei was scored as follows: 0=negative, no staining in cancer cells; 1=low, less than 25% of cancer cells stained positively for PROX1, intensity of staining was weak; 2=moderate, 25–50% of cancer cells were positive for PROX1; 3=strong, 50–75% of cancer cells were positive; 4=very strong, more than 75% of cancer cells were positive. If more than one tissue spot was available from the same patient, the highest score out of the parallel spots was selected for statistical analysis.

### Statistical analyses

The association between PROX1 immunohistochemistry results and clinicopathological variables was assessed by using the *χ*^2^ test. Life tables were computed according to the Kaplan–Meier method. Colorectal cancer-specific survival (CCSS) was calculated from the date of the diagnosis to death from CRC. Patients who died from causes other than CRC (87 out of 517) were censored on the date of death. Survival between the groups was compared using the logrank test. Multivariate survival analyses were carried out using the Cox proportional hazards model, and a *P*-value of 0.05 was adopted as the limit for inclusion of a covariate. All *P*-values are two-tailed.

## Results

### PROX1 expression in CRC

**Figure 1:**
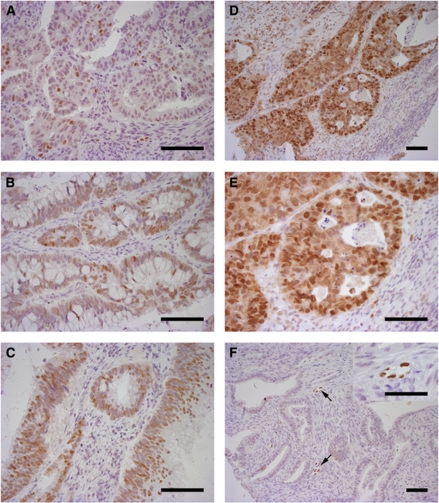
PROX1 expression in human colorectal cancer tissue microarray specimens. (**A**) Low PROX1 expression in colon cancer tissue; only few cancer cell nuclei are positive. (**B**) Moderate PROX1 expression in colon cancer tissue. (**C**) Strong PROX1 expression in rectal cancer tissue. (**D** and **E**) Very strong PROX1 expression in rectal cancer tissue. (**F**) Rectal cancer tissue negative for PROX1; however, adjacent lymphatic endothelial cells (arrow, insert) stained positively for PROX1. Scale bar=100 *μ*m.

Normal epithelium was mostly negative for PROX1, but the expression could be observed in a few crypt and neuroendocrine cells, and in the nuclei of lymphatic vessel endothelium beneath the mucosa. The majority of the tumour samples showed some degree of PROX1 expression (91%, 471 out of 517). Low PROX1 expression was detected in 24% (122 out of 517) of the tumours, moderate in 43% (224 out of 517), strong in 20% (105 out of 517), and very strong expression in 4% (20 out of 517). Representative immunostaining results are shown in Figure 1.

### Association between PROX1 expression and clinicopathological parameters

High PROX1 expression was significantly more frequent in high-grade (grade 3–4) tumours when compared with low-grade (grade 1–2) tumours (*P*=0.0001; Table 1). None of the grade 1 tumours had high PROX1 expression. No statistically significant association was found between PROX1 and age at diagnosis, tumour location, tumour site, gender, or Dukes stage (Table 1).

### Association of PROX1 expression with CCSS

**Figure 2:**
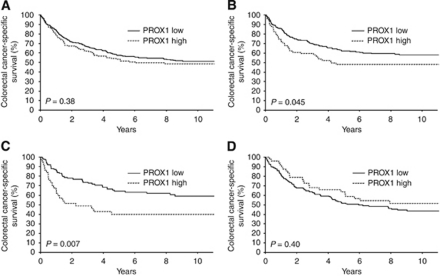
Disease-specific survival of 517 colorectal cancer patients according to PROX1 expression. (**A**) All colorectal cancer patients. ‘PROX1 low’, scores from 0 to 2, *n*=392; ‘PROX1 high’, scores 3 and 4, *n*=125. (**B**) Colon cancer patients. ‘PROX1 low’, *n*=218; ‘PROX1 high’, *n*=73. (**C**) Female colon cancer patients. ‘PROX1 low’, *n*=101; ‘PROX1 high’, *n*=36. (**D**) Rectal cancer patients. ‘PROX1 low’, *n*=174; ‘PROX1 high’, *n*=52.

For further analyses we categorised the patients into two groups, PROX1 low (staining scores from 0 to 2) and PROX1 high (scores 3 and 4). In the entire patient series, high PROX1 expression was not significantly associated with CCSS (RR=1.14; *P*=0.38; Table 2, Figure 2A). The 5-year CCSS was 57% (95% confidence interval (CI), 52.1–62.5%) among patients with low PROX1 expression level, and 53% (95% CI, 43.6–62.1%) when the PROX1 expression was high. In the subgroup of patients with colon cancer, high PROX1 expression was associated with unfavourable survival (RR=1.47; *P*=0.045; Table 2, Figure 2B). The 5-year CCSS of the colon cancer patients with low PROX1 expression was 62% (95% CI, 55.2–68.9%), as compared with 47% for those with high staining intensity (95% CI, 35.3–59.0%). The 5-year CCSS among colon cancer patients with very high (score 4; *n*=11) PROX1 expression was only 24%, suggesting that increased tumour cell expression of PROX1 is associated with worse outcome of the colon cancer patients. Furthermore, the 5-year CCSS of female colon cancer patients with low PROX1 expression (score 0–2) was 63% (95% CI, 53.3–73.0%), compared with 38% when the expression of PROX1 was high (95% CI, 22.0–54.7% RR=2.02, *P*=0.007; Figure 2C), whereas no significant difference was detected among male colon cancer patients (data not shown). No significant association was detected between PROX1 and survival in rectal cancer patients. The 5-year CCSS for PROX1 low patients was 51% (95% CI, 43.6–59.4%) and that PROX1 high patients was 61% (95% CI, 47.1–75.9% RR=0.82, *P*=0.4; Table 2, Figure 2D).

### Multivariate survival analysis

To adjust for established prognostic factors in colorectal cancer, PROX1 expression was entered into a Cox proportional hazards model together with Dukes stage, histological grade, age at diagnosis, tumour location, tumour site, and gender. In the multivariate survival analysis, PROX1 expression was not a significant prognostic factor (Table 3). Cox multivariate analysis was also performed for the subgroup of female colon cancer patients. However, PROX1 expression did not provide significant prognostic information in addition to the selected factors (data not shown).

## Discussion

To explore the clinical significance of PROX1, we investigated expression of PROX1 by immunohistochemistry in tissue microarray specimens of 517 patients with CRC. The present results indicate that high PROX1 expression is associated with the high grade of tumour differentiation and less favourable prognosis in the subgroup of patients with colon cancer. Moreover, our data show that high PROX1 expression is associated with unfavourable outcomes among the subset of female colon cancer patients. These observations are in line with the hypothesis that PROX1 overexpression promotes the progression of CRC (Petrova *et al*, 2008).

We found nuclear PROX1 expression in 91% of the 517 CRC specimens, and 27% of these samples showed a high level of expression. Currently, tumour grade is an important clinical indicator of prognosis in CRC and our study revealed that high PROX1 expression was associated with high tumour grade, but not with other clinicopathological parameters. A distinct level of differentiation may indicate different biological behaviour of the cancer. In the present study, none of the grade 1 tumours had high PROX1 expression. The reason for the lack of PROX1 expression in highly differentiated tumours remains unknown, but this could be due to the lower Wnt pathway activation. Additional studies are needed to address the mechanism of PROX1 action.

Previous studies have shown that PROX1 acts as a nuclear transcription factor (Oliver *et al*, 1993; Rodriquez-Niedenführ *et al*, 2001). On the basis of these studies and the preclinical findings by Petrova *et al* (2008), we chose to evaluate PROX1 expression according to the staining in tumour cell nuclei. However, it is also possible that PROX1 is enriched and/or activated in the cytoplasm before translocation into the nucleus to perform its biological function, and thus cytoplasmic PROX1 staining may be detectable. Regulation of intracellular localisation is momentous for transcription factor action, and nuclear import can serve as a mechanism to regulate gene expression (reviewed in Yoneda, 2000). In addition, it has been shown that Prospero, the *Drosophila* counterpart of PROX1, is often found in the cytoplasm of proliferating and undifferentiated cells (Li and Vaessin, 2000).

The current study shows that PROX1 has prognostic value among colon cancer patients, whereas no difference was found in rectal cancer patients (Table 2, Figure 2). Among colon cancer patients the difference in survival was evident in females and high PROX1 expression was associated with worse outcome. It has been suggested that prognosis for right-sided colon cancers is different from that for left-sided colon cancers (reviewed in Distler and Holt, 1997). Various reasons for such difference could include environmental factors, genetic factors, and sex distribution (reviewed in Iacopetta, 2002). During embryonic development, the right colon arises from the midgut and the left colon from the hindgut. Analyses of genetic databases from normal colon and tumour specimens have revealed differences in gene expression between normal mucosa and colon carcinomas originating from the right and left colons (Glebov *et al*, 2003; Birkenkamp-Demtroder *et al*, 2005). It was recently shown in a large population-based study that right-sided colon cancers have a worse prognosis than left-sided cancers and that women are more likely to get right-sided colon cancer, although women did not have significant difference in mortality between left- and right-sided colon cancers (Meguin *et al*, 2008). In the present study, the proportion of right-sided *vs* left-sided tumours was similar in males and females, and we found no significant difference in PROX1 expression between right-sided and left-sided tumours (data not shown).

As compared with other cancer types, very few molecular prognostic markers have been reported in CRC. Molecular markers for tumour tissue would be important for clinical decision making, because targeted therapy is an important goal for improving the outcomes of patients with CRC. To date, the exact mechanism of PROX1 action in normal and diseased tissue is poorly understood. Thus, further studies regarding the molecular mechanisms that regulate PROX1 expression and the direct transcriptional target genes of PROX1 are needed. In summary, our results show that high nuclear PROX1 expression is associated with unfavourable outcome in colon cancer patients, and in particular among female colon cancer patients. In addition, these results confirm the previous preclinical observations suggesting that PROX1 has a role in tumour progression in CRC.

## Figures and Tables

**Table 1:** Associations of PROX1 expression with clinicopathological variables

**Variable**	***n***	**PROX1 low, *n* (%)**	**PROX1 high, *n* (%)**	***P*-value *n* (%)** a
*Age*				0.4992
<50	60	50 (83)	10 (17)	
50–64	156	117 (75)	39 (25)	
65–74	174	132 (76)	42 (24)	
>75	127	93 (73)	34 (27)	
				
*Location*				0.5843
Rectum	226	174 (77)	52 (23)	
Colon	291	218 (75)	73 (25)	
				
*Site*				0.2069
Right	143	101 (71)	42 (29)	
Left	367	285 (78)	82 (22)	
Transverse colon	7	6 (86)	1 (14)	
				
*Gender*				0.8074
Male	282	215 (76)	67 (24)	
Female	235	177 (75)	58 (25)	
				
*Histological grade*				<0.0001
1	15	15 (100)	0 (0)	
2	347	276 (80)	71 (20)	
3	134	85 (63)	49 (37)	
4	20	15 (75)	5 (25)	
				
*Dukes stage*				0.2975
A	72	215 (76)	67 (24)	
B	193	152 (79)	41 (21)	
C	129	92 (71)	37 (29)	
D	123	90 (73)	33 (27)	

a *χ*^2^ test.

**Table 2:** Five-year CCSS of 516 patients with colorectal cancer according to nuclear PROX1 expression

**Factor**	**Score**	***n***	**5-year CCSS (95% CI)**	***P*-value** a	**RR**
All tumours					
	0–2	392	57.3 (52.2–62.4)		
	3–4	125	52.8 (43.8–61.8)	0.3769	1.14
					
*Age (years)*					
<50	0–2	50	56.4 (42.3–70.5)		
	3–4	10	80 (55.3–100.0)	0.1188	0.335
50–64	0–2	117	64.0 (55.4–72.6)		
	3–4	39	56.0 (39.7–72.3)	0.2426	1.381
65–74	0–2	132	59.7 (51.1–68.3)		
	3–4	42	45.2 (29.7–60.7)	0.1074	1.472
>75	0–2	93	45.8 (35.2–56.4)		
	3–4	34	50.7 (33.5–67.9)	0.6167	0.869
					
*Location*					
Rectum	0–2	174	51.5 (43.9–59.1)		
	3–4	52	61.5 (47.4–75.6)	0.4009	0.823
Colon	0–2	218	62 (55.3–68.7)		
	3–4	73	47.1 (35.5–58.7)	0.0451	1.474
					
*Site*					
Right	0–2	101	61.6 (51.8–71.4)		
	3–4	42	44.4 (29.1–59.7)	0.0705	1.602
Left	0–2	285	56.0 (50.1–61.9)		
	3–4	82	56.7 (45.5–67.9)	0.9912	1.002
Transverse colon	0–2	6	50.0 (10.0–90.0)		
	3–4	1	100 (NA)	NA	NA
					
*Gender*					
Male	0–2	215	56.8 (49.9–63.7)		
	3–4	67	61.7 (49.5–73.9)	0.5679	0.885
Female	0–2	177	57.9 (50.5–65.3)		
	3–4	58	42.7 (29.8–55.6)	0.0551	1.483
					
*Dukes stage*					
A	0–2	58	83.9 (74.3–93.5)		
	3–4	14	100.0 (100.0–100.0)	0.4129	0.434
B	0–2	152	78.1 (71.2–85.0)		
	3–4	41	81.2 (68.7–93.7)	0.2273	0.612
C	0–2	92	55.1 (44.5–65.7)		
	3–4	37	48.6 (31.9–65.3)	0.2021	1.391
D	0–2	90	7.1 (1.6–12.6)		
	3–4	33	6.1 (0–14.3)	0.8273	1.047
					
*Histological grade*					
1–2	0–2	291	60.6 (54.9–66.3)		
	3–4	71	61.8 (50.2–73.4)	0.9745	0.994
3–4	0–2	100	48.1 (37.9–58.3)		
	3–4	54	40.6 (26.9–54.3)	0.6056	1.125

Abbreviations: CCSS=cancer-specific survival; RR=relative risk.

a *χ*^2^ test.

**Table 3:** Cox multivariate regression of the association between PROX1 immunoreactivity and colorectal cancer-specific survival, adjusted for clinicopathological characteristics (*n*=516)

**Covariate**	**HR**	**95% CI**	***P*-value**
*PROX1 expression*			
Low	1.00		
High	0.908	0.672–1.227	0.5289
			
*Dukes stage*			
A	1.00		
B	1.712	0.882–3.323	0.1121
C	4.929	2.593–9.367	<0.0001
D	29.213	15.386–55.467	<0.0001
			
*Histological grade*			
1–2	1.00		
3–4	1.528	1.152–2.027	0.0033
			
*Age at diagnosis*			
<50 years	1.00		
50–64 years	1.595	0.986–2.582	0.0571
65–74 years	2.432	1.527–3.876	0.0002
>75 years	3.980	2.431–6.517	<0.0001
			
*Tumour location*			
Colon	1.00		
Rectum	1.601	1.158–2.213	0.0044
			
*Site*			
Right	1.00		
Left	0.783	0.539–1.137	0.1989
Transverse colon	1.731	0.617–4.855	0.2970
			
*Gender*			
Male	1.00		
Female	0.975	0.752–1.265	0.8480

Abbreviations: CI=confidence interval; HR=hazard ratio.
